# Effects of Lipoic Acid, Caffeic Acid and a Synthesized Lipoyl-Caffeic Conjugate on Human Hepatoma Cell Lines

**DOI:** 10.3390/molecules16086365

**Published:** 2011-07-27

**Authors:** Eliana Guerriero, Angela Sorice, Francesca Capone, Susan Costantini, Pasquale Palladino, Marco D'ischia, Giuseppe Castello

**Affiliations:** 1 “Pascale Foundation” National Cancer Institute - Cancer Research Center, Mercogliano (AV) Italy; 2 “Pascale Foundation” National Cancer Institute, Naples, Italy; 3 Department of Organic Chemistry and Biochemistry, University of Naples Federico II, Naples, Italy

**Keywords:** antioxidants, lipoic acid, caffeic acid, cytokines

## Abstract

Hepatocellular carcinoma (HCC) is among the most aggressive and fatal cancers. Its treatment with conventional chemotherapeutic agents is inefficient, due to several side effects linked to impaired organ function typical of liver diseases. Consequently, there exists a decisive requirement to explore possible alternative chemopreventive and therapeutic strategies. The use of dietary antioxidants and micronutrients has been proposed for HCC successful management. The aim of this work was to test *in vitro* the effects of lipoic acid, caffeic acid and a new synthesized lipoyl-caffeic conjugate on human hepatoma cell lines in order to assess their effect on tumor cell growth. The results of cytotoxicity assays at different times showed that the cell viability was directly proportional to the molecule concentrations and incubation times. Moreover, to evaluate the pro- or anti-inflammatory effects of these molecules, the cytokine concentrations were evaluated in treated and untreated cellular supernatants. The obtained cytokine pattern showed that, at the increasing of three molecules concentrations, three pro-inflammatory cytokines such as IL-1β, IL-8 and TNF-α decreased whereas the anti-inflammatory cytokine such as IL-10 increased.

## 1. Introduction

HCC is the sixth most common type of cancer in the World, with rates increasing from year to year and its incidence is mainly correlated with chronic hepatitis B and C virus infection and liver cirrhosis [1,2,3,4]. HCC treatment with conventional chemotherapeutic agents is inefficient, due to several side effects linked to impaired organ function typical of liver disease; in fact these therapies offer a probability of 5-year survival of 50–70% [5]. Therefore, the limited effectiveness of chemotherapy and high recurrence rate highlights the urgent need to identify new molecular targets and to develop new treatments. Numerous epidemiological studies have recently highlighted the existence of an inverse association between fruits and vegetables consumption, natural antioxidants and cancer risk; in fact, the antioxidants intake through diet or supplements of plant origin is strongly recommended for cancer prevention and cure [6]. In general, the antioxidants are substances of vegetable or mineral or animal origin that neutralize free radicals and protect the body from their negative actions on the plasma membrane, proteins and DNA [7,8,9]. In particular, they can prevent cancer by interfering with its early processes and, at the same time, stimulating the immune system to destroy cancer cells and to block their proliferation. Some studies on mouse models have shown that antioxidants are able to promote regression of several types of cancer [10]. In this work we have studies the effects of natural antioxidants on human hepatoma cell lines and focused the attention on lipoic and caffeic acid because both are present in many animal species and vegetables. Lipoic acid was first isolated in 1951 from bovine liver extracts [11] and exists in an open (DHLA) or close (LA) state in which two sulfhydryl groups, in position 6 and 8, are interconvertible by redox reactions [12,13]. It is a cofactor of several enzymes involved in the conversion process of glucose, and fatty acids into ATP, and, thus, is involved in the Krebs cycle. Caffeic acid (3,4-dihydroxycinnamic acid) is so-named because it was originally found in the coffee extracts [14]. Its antioxidant power depends by redox reactions of two hydroxyl groups of phenolic ring. The beneficial antioxidant properties of caffeic acid have been demonstrated both in vivo and in vitro [15]. Since the use of dietary antioxidants has been proposed as effective against some cancers and, also, HCC, we have synthesized a new antioxidant molecule, namely 2-S-lipoyl-caffeic acid, by condensation of sulfur atom of reduced form of lipoic acid (DHLA) with phenolic ring of caffeic acid, in order to try to improve the antioxidant activity of two natural molecules, and to reduce the proliferation of hepatoma cells.

## 2. Results and Discussion

### 2.1. 2-S-lipoyl-caffeic acid synthesis

**Figure 1:**
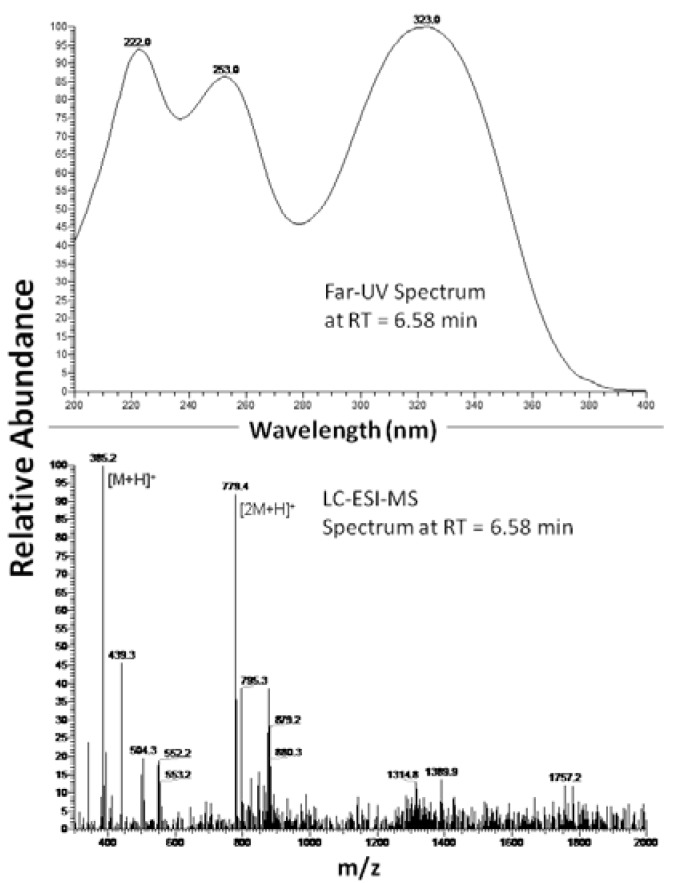
Far-UV spectrum and spectrum ESI.

Lipoic acid was reduced to dihydrolipoic acid with sodium borohydride (NaBH_4_) for 10 minutes at room temperature in doubly distilled water deoxygenated by purging with argon gas. Residual NaBH_4_ was eliminated by acidification with HCl solution [16]. Caffeic acid (3,4-dihydroxycinnamic acid) was oxidized to its *ortho*-quinone form in CH_3_CN at room temperature, purged with argon gas, with polymer-supported periodate in three minutes [17]. Subsequently, the periodate resin was quickly filtered off and the caffeic acid *ortho*-quinone solution was added to dihydrolipoic acid solution. The formation of 2-S-lipoyl-caffeic acid was monitored by UV-visible spectroscopy and liquid chromatography-mass spectrometry (LC-ESI-MS) [18]. Figure 1 shows the UV-VIS spectrum of 2-S-lipoyl-caffeic acid taken at HPLC peak apex at retention time (RT) 6.58 minutes, showing three maxima at 222, 253 and 323 nm recognized as characteristic of 2-S-thionyl-caffeic acid adduct [19]. LC-ESI-MS analysis of reaction product at retention time (RT) 6.58 minutes reported in Figure 1 showed two predominant experimental peaks with *m/z* values equal to 385.2 and 779.4, confidently assigned to 2-S-lipoyl caffeic acid mass and double mass, respectively, although slightly different from the theoretical molecular weight 386.09. However these differences between the experimental and theoretical masses are due to small calibration error of LC-ESI-MS instrument.

### 2.2. Colorimetric assay with Sulforhodamine B

**Figure 2:**
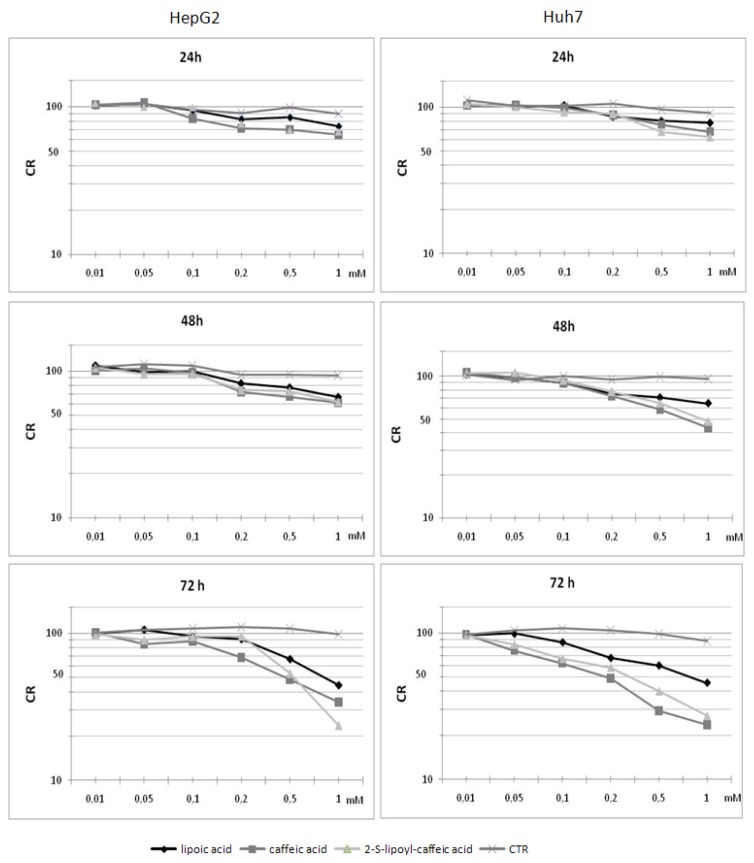
HepG2 and Huh7 cell lines growth curves in the absence and presence of lipoic acid, caffeic acid and 2-S-lipoyl-caffeic acid. On the x-axis is showed the different molecular concentrations (mM), on the y-axis cell growth rate (CR).

The cell viability was determined after 24, 48 and 72 h stimulation by three antioxidants in HepG2 and Huh7 to identify the IC_50_ concentration, corresponding to the chemical concentration that causes 50% inhibition of cell growth (Figure 2). The cellular viability of untreated cells was used as control. 

### 2.3. Cytotoxicity assay on HepG2 cells

HepG2 cells showed a good level of viability after 24 and 48 h treatment with different concentrations of lipoic acid, caffeic acid and 2-S-lipoyl-caffeic acid; in fact, there was a reduction in cell viability but not significant, as evidenced by the overlapping of growth curves in Figure 2. The decrease in cell viability became significant after 72 h incubation concerning 0.5 mM concentration for both caffeic acid and 2-S-lipoyl-caffeic acid and 0.8 mM for lipoic acid (Figure 2; Table 1S in Supplementary Material). However, caffeic acid after 72 h was more effective than lipoic acid, whereas it showed similar efficacy respect to 2-S-lipoyl-caffeic acid. 

### 2.4. Cytotoxicity assay on Huh7 cells

After 24 h treatment with all three antioxidants the cytotoxicity assay on Huh7 cell line showed no significant inhibitory effects on cell proliferation, while after 48 h treatment with 0.8 mM caffeic acid and 1 mM 2-S-lipoyl-caffeic acid it caused an significant inhibition of tumor cellular growth (Table 2S in Supplementary Material). The IC_50_ corresponding to cell viability reduction equal to 50–80% compared to control cells was obtained after 72 h incubation with lipoic acid, caffeic acid and 2-S-lipoyl-caffeic acid using the 0.75, 0.2 and 0.3 mM doses, respectively (Figure 2). Therefore, caffeic acid, as for HepG2, showed a greater cytotoxic effect than lipoic acid. 

### 2.5. Bio-Plex assay

**Figure 3:**
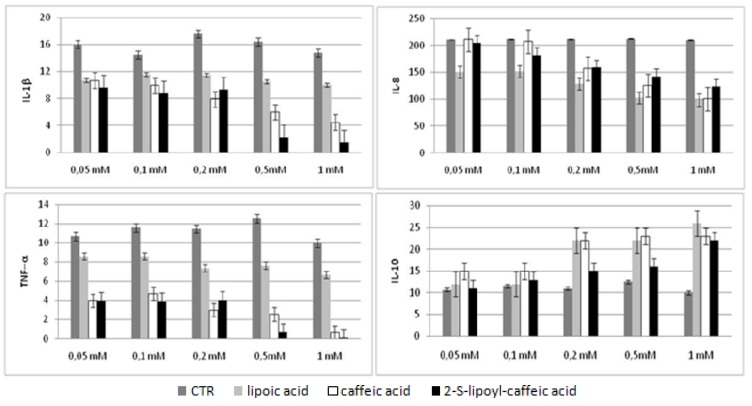
Cytokines levels in HepG2 cell line after 72 h of treatment.

**Figure 4:**
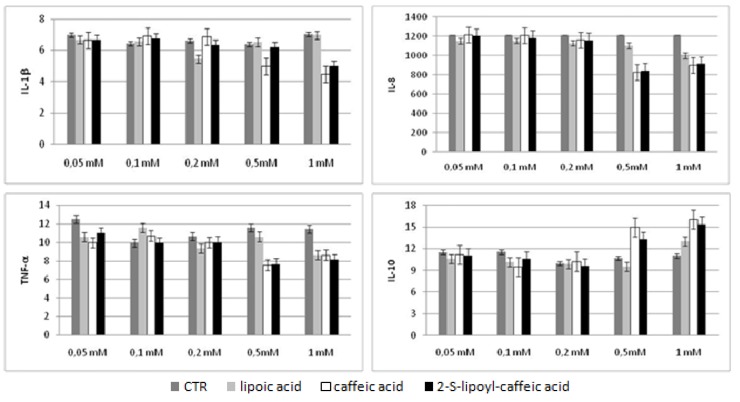
Cytokines levels in Huh7 cell line after 48 h of treatment.

**Figure 5:**
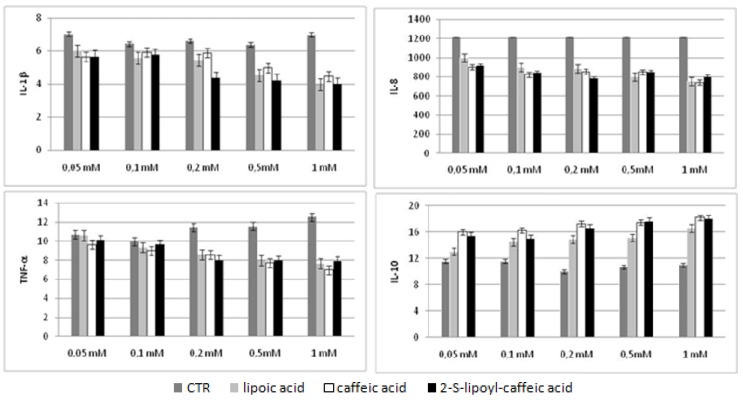
Cytokines levels in Huh7 cell line after 72 h of treatment.

We evaluated the cytokines production in HepG2 cellular supernatants after incubation with three molecules at 72 h and in Huh7 after incubation at 48 and 72 h by Bio-Plex assay. The results obtained were compared with untreated cells used as control. These experiments showed that the levels of three pro-inflammatory cytokines, like TNF-α, IL-8 and IL-1β, decreased in statistically significant way at concentration increasing of caffeic acid, lipoic acid and 2-S-lipoyl-caffeic acid. However, this decrease was dependent dose in both HepG2 and Huh7 treated at 72 h, whereas in Huh7 treated at 48 h a significant reduction was observed only using 0.5 mM caffeic acid and 1 mM 2-S-lipoyl-caffeic acid in agreement with the results of cytotoxicity test (Figure 3, Figure 4, Figure 5). Moreover, IL-10 being anti-inflammatory cytokine showed a statistically significant increase in Huh7 cells treated at 48 h using 0.5 mM caffeic acid and 1 mM 2-S-lipoyl-caffeic acid, and in HepG2 and Huh7 treated at 72 h using increasing dose of all three antioxidants (Figure 3, Figure 4, Figure 5).

### 2.6. Discussion

The use of antioxidants may be useful tool to contrast or at least to balance the tumor growth, so they could represent an effective resource in anticancer therapeutic strategies. Two natural antioxidants molecules (*i.e.*, lipoic acid, and caffeic acid), tested in this work, highlighted good potential as inhibitors of tumor cell growth in vitro with dose and time dependent toxic effect. This was in agreement to previous papers that showed a significant decrease in cell proliferation of HepG2 cells after 72 h of incubation with lipoic acid using the 100–500 μM concentrations [20] and the reduction in cell viability of HepG2 after stimulation with 200 μg/mL caffeic acid for 24 h [21].

Moreover, the caffeic acid role in anticancer prevention strategies has been recently investigated in Huh7 cells that, treated with CADPE, a caffeic acid derivate, showed an inhibition of the expression levels of cyclin D1, an important regulator of cell cycle that promotes the proliferation and tumor growth [22,23]. 

Our data also evidenced that Huh7 cells were more sensitive to antioxidants than HepG2 cells, reaching IC_50_ after 48 h of treatment. These results could depend from the fact that Huh7 cells are more undifferentiated respect to HepG2. In fact, recent studies have shown that the Huh7 cell line is associated with low expression of cytokeratin 8/18 (CK8/18), while HepG2 cell line is correlated with high expression of CK8/18 being usually expressed in normal hepatocytes [24]. In addition, HepG2 cells express p53 in their native form while Huh7 cells constitutively express the mutated form of the same protein and, therefore, are characterized by a more malignant phenotype [25]. 

To try to enhance the positive effects of these antioxidants we have synthesized a new conjugated molecule, namely 2-S-lipoyl-caffeic acid, that resulted to have a cytotoxicity level similar to caffeic acid, and this could be due to the presence in the newly synthesized molecule of three hydroxyl groups present in caffeic acid. 

Since in HCC the inflammation persistence resulted to play an important role in the transition from a chronic liver disease to neoplastic process [26,27] we also studied the immunomodulatory role of these three antioxidants on the production of cytokines in hepatoma cellular supernatants by a multiplex biometric ELISA-based immunoassay. In literature it is reported that HepG2 cells expressed mRNAs of various cytokines including IL-8, IL-10 and TNF-α [28,29]. On the other hand, studies on HCC patients, conducted in our laboratory, showed that high levels of IL-8 correlated with tumor size suggesting that this interleukin could have a role in the HCC progression and could be considered a marker of tumor invasiveness [26]. Our results showed that lipoic acid, caffeic acid and 2-S-lipoyl-caffeic acid induced the decreasing of pro-inflammatory cytokines such as TNF-α, IL-1β and IL-8 and the increasing of anti-inflammatory cytokines such as IL-10. This suggested that these three molecules can promote tumor regression and inhibit tumor invasiveness. In particular, TNF-α is a pro-inflammatory cytokine that plays an important role in apoptosis, proliferation, in cell differentiation and viral replication and the increase of its expression is involved in many diseases and cancers [30]. Its high levels are known to induce in HCC the activation of anti-apoptotic transcription factor NF-kB, that promotes cell survival and tumor progression [31,32]. Therefore, a reduction of TNF-α, as found in this study, could indicate that it anticipates the activation of NF-kB by blocking tumor growth. This agreed to other papers reporting that lipoic acid and caffeic acid inhibited in a dose-dependent way the activation of NF-kB induced by TNF-α, both in human U937 histocyte cells and in human aortic endothelial cells [33,34] and that caffeic acid can reduced the TNF-α expression in gastric epithelial cells stimulated with *Helicobacter pylori* [35], in RAW264,7 cells [36] and endothelial cells of the middle ear HMEECs [37]. 

Moreover, IL-8 is a pro-inflammatory chemokine having a strong pro-angiogenic activity in HCC patients [38] and its expression in HepG2 cells has been correlated with invasiveness and tumor metastasis because it increased more in the later stages of HCC [39]. IL-8 reduction obtained after the treatment of HepG2 and Huh7 cells with lipoic acid, caffeic acid and 2-S-lipoyl-caffeic acid, suggested that it could inhibit the tumor invasiveness promoting cellular regression in according to some studies on caffeic acid that evidenced that this molecule reduced its expression in endothelial cells HUVEC [40], in Caco-2 cells [41], and in SW620 colon epithelial cells [42]. 

On the other hand IL-1β promotes inflammatory processes by stimulating the production of prostaglandins and other cytokines and its reduction, obtained in our work, was consistent with a published work which showed significant reduction of IL-1β in HepG2 and Huh7 treated with 25 μg/mL of caffeic acid (CAPE) [43].

Finally, IL-10 has a direct effect on apoptosis by blocking the activation of NF-kB, and its increase could lead to programmed cell death of tumor cells as already demonstrated in a study conducted on primary cultures of rat hepatocytes and HepG2 cell line [44] and in another studies on HCC patients in the later stages of disease [45,46].

## 3. Experimental

### 3.1. General

Lipoic and caffeic acid were purchased from VWR International (Leuven, Belgium), HPLC chemicals were purchased from Lab-Scan (Dublin, Ireland), other chemicals were purchased from Sigma Aldrich (St. Louis, MO, USA) and the columns for purification and characterization were purchased from Phenomenex (Torrance, CA, USA). Product purity and integrity was confirmed by LC–ESI-MS (Finnigan Surveyor, Thermo Electron Corporation) applying a linear gradient of acetonitrile/0.05% TFA in water/0.05% TFA from 40 to 95% over 13 min.

### 3.2. Cell culture

Human hepatoma cell lines (HepG2, Huh7) were kept in culture and expanded at 37 °C in a humidified atmosphere of 5% CO2 in culture medium DMEM (Dulbecco's Modified Eagle's Medium, Lonza, Verviers, Belgium), supplemented with FBS (Invitrogen, Camarillo, CA, USA) at 10%, Penicillin/Streptomycin 100× (Euroclone, Devon, UK), Glutamax 100× (Invitrogen) and non-essential amino acids 100× (Invitrogen). Phosphate buffer (PBS phosphate buffered saline Ca^2+^ and Mg^2+^ free) and trypsin (Ca^2+^ and Mg^2+^ free) were supplied by Euroclone.

### 3.3. Colorimetric assay with Sulforhodamine B

Cell proliferation was assessed in presence and absence of lipoic acid, caffeic acid and 2-S-lipoyl-caffeic acid by colorimetric assay with sulforhodamine B (SRB, Sigma Aldrich) [47]. The cells (2 × 10^4^) were seeded in 96-multiwell plates in 200 μL of culture medium, and left to grow for 24 h at 37 °C for allowing adhesion. Then, the cells were treated with these molecules using different concentrations (0.01 mM, 0.05 mM, 0.1 mM, 0.2 mM, 0.5 mM and 1 mM) and incubated for 24, 48 and 72 h. All the antioxidants were dissolved in dimethyl sulfoxide (DMSO, Sigma Aldrich) at a concentration of 100 mM. In cell cultures the DMSO concentration remained always below 0.1%, a dose that did not exert toxic effects [48]. Then, the cells were fixed for 1h at 4 °C with 50% trichloroacetic acid (TCA, Sigma Aldrich). Subsequently, the cells were washed with distilled water and were dried to air. 100 µL of SRB was added to each well and the plate was incubated for 30 min at RT. To remove the dye excess the cells were washed using 1% acetic acid. The number of viable cells was directly proportional to the protein bound-dye formation which was then solubilized with 10 mM Tris base solution pH 10.5 and measured by fluorometric assay ELISA at 540 nm (Bio-Rad, Hercules, CA, USA; Microplate Reader). All experiments were performed in duplicate and were repeated for three times. The cellular viability was estimated as % compared to untreated cells.

### 3.4. Bio-Plex assay

A multiplex biometric ELISA-based immunoassay, containing dyed microspheres conjugated with a monoclonal antibody specific for a target protein was used, according to the manufacturer’s instructions (Bio-Plex, Bio-Rad), to evaluate the trends and the concentrations of different cytokines after 24, 48 and 72 h of incubation with the molecules in the cellular supernatants. Soluble molecules were measured using an Ultrasensitive Human Cytokine 10-Plex Panel (Invitrogen, Corporation, 542 Flynn Rd, Camarillo, CA). In particular, the following cytokines were evaluated: GM-CSF, IL-1β, IL-2, IL-4, IL-5, IL-6, IL-8, IL-10, IFNγ and TNF-α. Each experiment was performed in duplicate as previously described [26,27]. Protein concentrations were determined using a Bio-Plex array reader (Luminex, Austin, TX) and the analyte concentration was calculated using a standard curve, with software provided by the manufacturer (Bio-Plex Manager Software). 

### 3.5. Statistical analysis

The cytokines concentrations evaluated in treated *vs.* untreated cell lines were compared by T-test. Values of p < 0.05 were considered to be statistically significant. The statistical program Prism 4 (GraphPad Software, San Diego, CA, USA) was used.

## 4. Conclusions

HCC treatment with conventional chemotherapeutic agents is inefficient, due to several side effects linked to impaired organ function typical of liver diseases. Therefore, it needs to explore possible alternative chemopreventive and therapeutic strategies. In fact, the use of dietary antioxidants and micronutrients has been recently proposed for HCC successful management [7,8,9]. For these reasons, in this work we have synthesized lipoyl-caffeic conjugate and tested *in vitro* the effects of lipoic acid, caffeic acid and new molecule on human hepatoma cell lines. Moreover, we have evaluated the cytokine concentrations in treated and untreated cellular supernatants in order to evaluate the pro- or anti-inflammatory effects of three molecules. In conclusion our data evidenced that (a) these antioxidants inhibited the tumor cell growth in dose and time dependent way, (b) they induced the TNF-α, IL-1β and IL-8 decrease and the IL-10 increase promoting tumor regression and (c) new molecule 2-S-lipoyl-caffeic acid had a cytotoxic effects similar to caffeic acid. Therefore, further studies will regard the design and the synthesis of new molecules containing a different number of hydroxyl groups respect to caffeic acid, and blocking the second SH group present in lipoic acid.

## Supplementary Materials

Supplementary File 1
