# Supplementary material for: Effects of Lipoic Acid, Caffeic Acid and a Synthesized Lipoyl-Caffeic Conjugate on Human Hepatoma Cell Lines
Source: Molecules. 2011 Jul 27;16(8):6365–77. doi: 10.3390/molecules16086365 (PMC6264529; doi:10.3390/molecules16086365)
Supplement: Supplementary File 1 [file molecules-16-06365-s001.pdf]

Supplementary Material

Table 1S. Cytotoxicity assay results performed on HepG2 cells after treatment with lipoic acid, caffeic acid and 2-S-lipoil-caffeic acid at 24, 48 and 72 h.

	Lipoic Acid	***Caffeic Acid***	***2-S-lipoil-caffeic acid***
HepG2 24 h	No effect on cell viability	No effect on cell viability	No effect on cell viability
HepG2 48 h	No significant reduction on cell viability	No significant reduction on cell viability	No significant reduction on cell viability
HepG2 72 h	IC50 = 0.8 mM; ***60%*** reduction on cell viability at 1 mM	IC50 = 0.5 mM; ***70%*** reduction on cell viability at 1 mM	IC50 = 0.5 mM; ***80%*** reduction on cell viability at 1 mM

Table 2S. Cytotoxicity assay results performed on Huh7 cells after treatment with lipoic acid, caffeic acid and 2-S-lipoil-caffeic acid at 24, 48 and 72 h.

	Lipoic Acid	***Caffeic Acid***	***2-S-lipoil-caffeic acid***
Huh7 24 h	No effect on cell viability	No effect on cell viability	No effect on cell viability
Huh7 48 h	No significant reduction on cell viability	IC50 = 0.8 mM	IC50 = 1 mM
Huh7 72 h	IC50 = 0.75 mM	IC50 = 0.2 mM; ***80%*** reduction on cell viability at 1 mM	IC50 = 0.3 mM; ***70%*** reduction on cell viability at 1 mM.
